# Identification of a blood-borne miRNA signature of synovial sarcoma

**DOI:** 10.1186/s12943-015-0424-z

**Published:** 2015-08-07

**Authors:** Alba Fricke, Prisca V. Ullrich, Jürgen Heinz, Dietmar Pfeifer, Jutta Scholber, Georg W. Herget, Oliver Hauschild, Peter Bronsert, G. Björn Stark, Holger Bannasch, Steffen U. Eisenhardt, David Braig

**Affiliations:** Department of Plastic and Hand Surgery, University Medical Center Freiburg, Hugstetter Strasse 55, 79106 Freiburg, Germany; Department of Hematology, Oncology and Stem Cell Transplantation, University Medical Center Freiburg, Hugstetter Strasse 55, 79106 Freiburg, Germany; Department of Radiation Oncology, University Medical Center Freiburg, Robert-Koch-Straße 3, 79106 Freiburg, Germany; Department of Orthopaedics and Traumatology, University Medical Center Freiburg, Hugstetter Strasse 55, 79106 Freiburg, Germany; Institute for Surgical Pathology, University Medical Center Freiburg, Breisacher Str. 115a, 79106 Freiburg, Germany; Tumorbank Comprehensive Cancer Center Freiburg, Breisacher Str. 115a, 79106 Freiburg, Germany

**Keywords:** Whole-blood-RNA, miRNA, Synovial sarcoma, Biomarker, Liquid biopsy

## Abstract

**Background:**

Synovial sarcoma account for approximately 10 % of all soft-tissue tumors and occur most frequently in young adults. A specific translocation in this sarcoma induces fusion of the SYT gene on chromosome 18 to the SSX genes on chromosome X, leading to proliferation of the tumor cells. The need for non-invasive biomarkers indicating recurrence and activity of this disease has sparked research into short non-coding RNA known as microRNA (miRNA).

**Methods:**

Blood samples of patients with active synovial sarcoma and of synovial sarcoma patients in complete remission as well as of healthy donors and patients with active leiomyosarcoma, MPNST, Ewing sarcoma and liposarcoma were collected. Whole blood RNA was extracted and samples of patients with active synovial sarcoma and of healthy donors were analyzed using an Affymetrix GeneChip miRNA Array v. 4.0. qRT-PCR was carried out to confirm a panel of miRNAs which where differentially expressed in the miRNA array. This miRNA-panel was further evaluated in patients with synovial sarcoma in complete remission and patients with active leiomyosarcoma, MPNST, Ewing sarcoma and liposarcoma as well as in an independent cohort of synovial sarcoma patients.

**Results:**

Unsupervised hierarchical clustering of the miRNA arrays separated patients with active synovial sarcoma from healthy controls. A panel of seven miRNAs (miR-99a-5p, miR-146b-5p, miR-148b-3p, miR-195-5p, miR-223-3p, miR-500b-3p and miR-505-3p) was further validated by qRT-PCR to be significantly upregulated in synovial sarcoma patients. Moreover, most of the analyzed miRNAs were shown to be significantly upregulated in synovial sarcoma patients compared to leiomyosarcoma, MPNST, Ewing sarcoma and liposarcoma patients. Validation of the miRNA panel in an independent cohort of synovial sarcoma patients confirmed higher expression levels compared to healthy controls and patients in complete remission.

**Conclusion:**

Our results have identified a specific whole blood miRNA signature that may serve as an independent biomarker for the diagnosis of local recurrence or distant metastasis of synovial sarcoma. It even distinguishes synovial sarcoma from other sarcoma subtypes, thus potentially serving as a specific biomarker for synovial sarcoma.

**Electronic supplementary material:**

The online version of this article (doi:10.1186/s12943-015-0424-z) contains supplementary material, which is available to authorized users.

## Background

Soft tissue sarcoma constitute a heterogeneous group of malignant tumors of mesenchymal origin, showing frequent local recurrence and distant metastasis [1, 2]. Synovial sarcoma account for approximately 10 % of all soft-tissue sarcoma and most frequently develop in the extremity of young adults [3]. The cytogenetically defined translocation t(X; 18) (p11.2; q11.2) found in synovial sarcoma results in the fusion of the SYT gene on chromosome 18 to either SSX1, SSX2 or SSX4 on chromosome X, resulting in the formation of a SS18-SSX1, SS18-SSX2 or the rare SS18-SSX4 fusion transcript [4–7], which allow a very specific and sensitive molecular diagnosis of synovial sarcoma by fluorescence *in situ* hybridization (FISH) or quantitative and conventional reverse transcription-polymerase chain reaction (RT-PCR) of tumor tissue [8]. However, diagnosis of local recurrence or distant metastasis of synovial sarcoma is restricted to magnetic resonance imaging (MRI), computed tomography (CT) and biopsy [2]. Therefore, the need for non-invasive biomarkers indicating recurrence and activity of this disease has sparked research into microRNAs (miRNAs), which are small, non-coding molecules of about 22 nucleotides in length. MiRNAs regulate the expression of target genes through mRNA degradation and translation inhibition [9], and have been shown to serve as potential biomarkers in different malignancies [10–12].

**Table 1:** MiRNAs with a fold change of > |3.0|, a *p*-value <0.005, a q-value <0.2 and a similar deregulation of related miRNAs with the same base sequence throughout the array

Transcript ID	Sarcoma	Sarcoma	Control	Control	Fold Change
	Mean	STD	Mean	STD	
*upregulated*					
hsa-miR-146b-5p	4.83	0.85	1.81	0.65	8.10
hsa-miR-99a-5p	4.51	0.35	1.92	1.17	6.03
hsa-miR-223-3p	10.50	0.66	8.25	0.91	4.77
hsa-miR-148b-3p	6.68	0.82	4.58	0.74	4.29
hsa-miR-500b-3p	4.28	0.65	2.28	0.82	4.01
hsa-miR-183-3p	6.83	0.46	4.97	0.90	3.62
hsa-miR-589-5p	4.84	0.57	3.06	0.82	3.43
hsa-miR-505-3p	3.73	0.67	1.96	0.61	3.40
hsa-miR-195-5p	2.96	0.43	1.24	0.46	3.30
*downregulated*					
hsa-miR-1225-5p	4.76	0.64	6.89	0.64	−4.39

Interestingly, many poorly differentiated tumors can be successfully classified using miRNA expression profiles whereas messenger RNA profiles may be rather inaccurate when applied to the same samples [13]. Recent publications show that sarcoma tissue of different histological subtypes show distinct miRNA expression profiles [14–16]. However, few studies concentrate on peripheral blood miRNA profiling. Miyachi et al. detected miR-206 to be significantly upregulated in peripheral blood of patients with rhabdomyosarcoma compared to patients with non-rhabdomyosarcoma tumors [17], thus showing that miRNA expression levels may be used to differentiate between different tumors. Furthermore, the detection of miRNAs in peripheral blood may serve as a non-invasive method for diagnosis of metastasis or local tumor recurrence, possibly improving survival rates due to earlier detection of sarcoma recurrence as well as making follow-up more economical. Therefore, we performed a comprehensive microarray-based miRNA screen of whole blood RNA of synovial sarcoma patients compared to healthy controls, followed by validation of deregulated miRNAs using quantitative Real-Time polymerase chain reaction (qRT-PCR), assessing differences in patients with active synovial sarcoma compared to healthy controls, patients with synovial sarcoma in complete remission and patients with active leiomyosarcoma, malignant peripheral nerve sheath tumor (MPNST), Ewing sarcoma and liposarcoma.

## Results and discussion

**Fig. 1:**
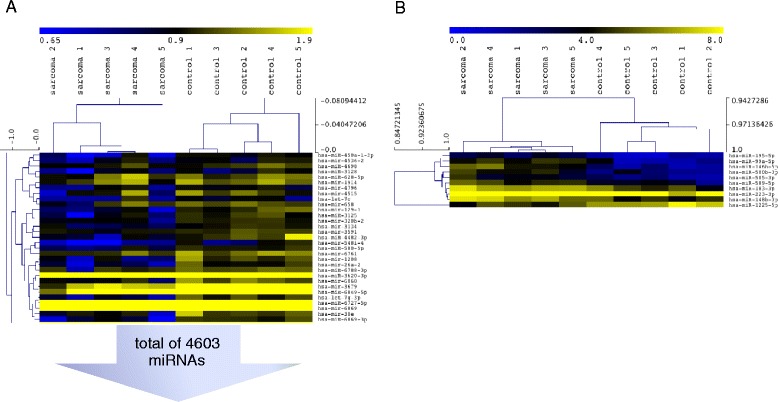
Hierarchical clustering separated sarcoma samples from control samples. **a**. Hierarchical clustering of all covered human mature miRNAs and human pre-miRNAs. **b**. Hierarchical clustering of the 10 miRNAs meeting the following criteria: Fold change > |3.0|, p-value <0.005, q-value <0.2 and a similar deregulation of related miRNAs with the same base sequence throughout the array

The screening cohort of patients with active synovial sarcoma (n = 5) as analyzed in a microarray as well as the independent validation cohort of synovial sarcoma patients (n = 3) did not differ significantly from the healthy controls (n = 5), synovial sarcoma in complete remission (n = 10) and active leiomyosarcoma (n = 5), MPNST (n = 3), Ewing sarcoma (n = 3) and liposarcoma (n = 6) group concerning age, BMI, hemoglobin (Hb) level, platelet count and leukocyte count (Additional file 1 and Additional file 2). Two patients with active synovial sarcoma presented the SS18-SSX2 fusion gene phenotype, while five presented the more common SS18-SSX1 phenotype [6, 18]. Tumor tissue of one patient was not available for analysis. Information regarding disease and therapy status of sarcoma patients is depicted in Additional file 3. Whole blood RNA from patients with active synovial sarcoma (n = 5) and healthy controls (n = 5) was analyzed by Affymetrix miRNA 4.0 Arrays. Microarray data are available in the ArrayExpress database (www.ebi.ac.uk/arrayexpress) under accession number E-MTAB-3273 (http://www.ebi.ac.uk/arrayexpress/experiments/E-MTAB-3273). Hierarchical clustering of all covered human mature miRNAs and human pre-miRNAs separated sarcoma samples from control samples (Fig. 1a). Following hierarchical clustering, the array results were narrowed down to deregulated miRNAs with a fold change > |3.0|, a p-value <0.005, a q-value (false discovery rate (FDR)-corrected p-value) [19] <0.2 and a similar deregulation of related miRNAs with the same base sequence throughout the array. Of the ten miRNAs, which met these criteria (miR-99a-5p, miR-146b-5p, miR-148b-3p, miR-183-3p, miR-195-5p, miR-223-3p, miR-500b-3p, miR-505-3p, miR-589-5p and miR-1225-5p), nine miRNAs showed an increased expression in tumor patients and one miRNA was decreased (Table 1). Unsupervised hierarchical clustering of this panel could again separate sarcoma patients from healthy controls (Fig. 1b).

**Fig. 2:**
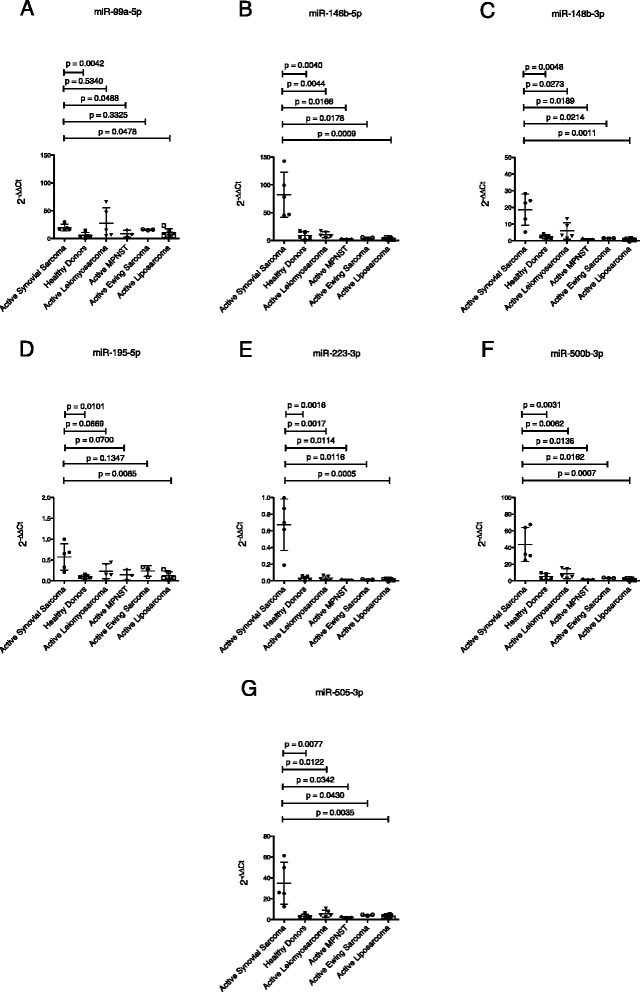
MiRNA-expression in patients with active synovial sarcoma compared to healthy donors and patients with active leiomyosarcoma, MPNST, Ewing sarcoma and liposarcoma. **a**. miR-99a-5p. **b**. miR-146b-5p. **c**. miR-148b-3p. **d**. miR-195-5p. **e**. miR-223-3p. **f**. miR-500b-3p. **g**. miR-505-3p

**Fig. 3:**
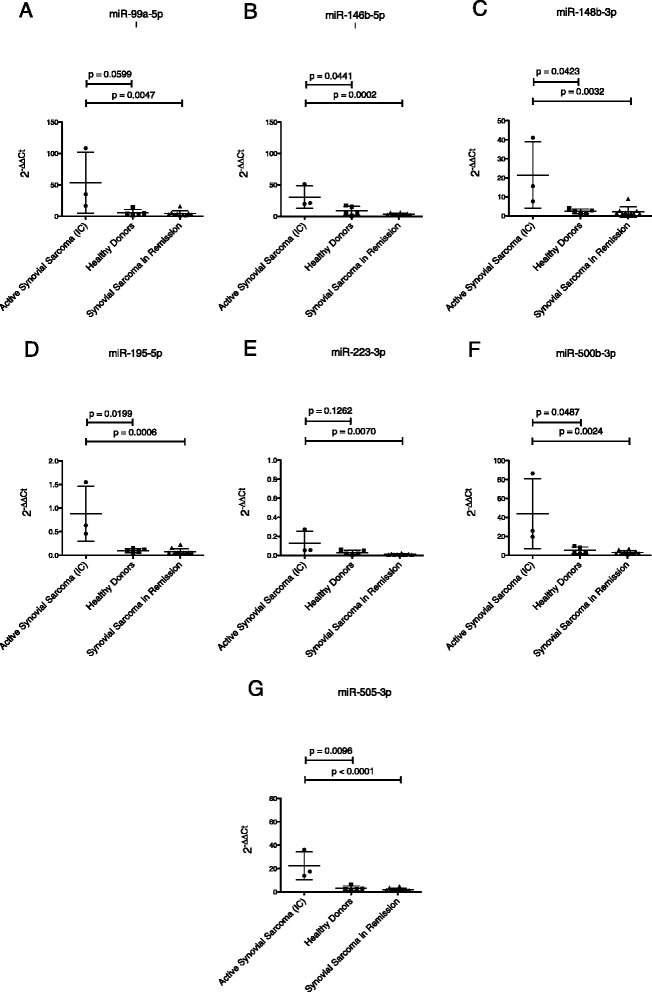
MiRNA-expression in the independent cohort (IC) of patients with active synovial sarcoma compared to healthy donors and patients with synovial sarcoma in remission. **a**. miR-99a-5p. **b**. miR-146b-5p. **c**. miR-148b-3p. **d**. miR-195-5p. **e**. miR-223-3p. **f**. miR-500b-3p. **g**. miR-505-3p

Subsequently, seven miRNAs were further validated by qRT-PCR (Fig. 2): miR-99a-5p, miR-146b-5p, miR-148b-3p, miR-195-5p, miR-223-3p, miR-500b-3p and miR-505-3p. All seven miRNAs were significantly upregulated in the screening cohort of patients with synovial sarcoma (n = 5) compared to healthy controls (n = 5). Moreover, five of the analyzed miRNAs were shown to be significantly upregulated in the screening cohort of active synovial sarcoma patients compared to patients with active leiomyosarcoma (n = 5): miR-146b-5p, miR-148b-3p, miR-223-3p, miR-500b-3p and miR-505-3p. MiR-99a-5p and miR-195-5p were not significantly deregulated (Fig. 2). Comparing the screening cohort of active synovial sarcoma patients to patients with active MPNST (n = 3), six of the analyzed miRNAs were shown to be significantly upregulated: miR-99a-5p, miR-146b-5p, miR-148b-3p, miR-223-3p, miR-500b-3p and miR-505-3p. MiR-195-5p was not significantly deregulated (Fig. 2). Analyzing active synovial sarcoma patients and patients with active Ewing sarcoma (n = 3), five of the analyzed miRNAs were shown to be significantly upregulated: miR-146b-5p, miR-148b-3p, miR-223-3p, miR-500b-3p and miR-505-3p. MiR-99a-5p and miR-195-5p were not significantly deregulated. Compared to patients with active liposarcoma (n = 6), all seven analyzed miRNAs were shown to be significantly upregulated (Fig. 2). These results suggest that the detection of these miRNAs in whole blood of synovial sarcoma patients provides a non-invasive way to detect distant metastasis or local recurrence and this “liquid biopsy” could potentially develop into a further keystone of tumor diagnosis and staging. We therefore validated the expression levels of all seven miRNAs by qRT-PCR in an independent cohort of active synovial sarcoma patients (n = 3): Five of the analyzed miRNAs were shown to be significantly upregulated when compared to the group of healthy donors (miR-146b-5p, miR-148b-3p, miR-195-5p, miR-500b-3p and miR-505-3p). MiR-99a-5p and miR-223-3p were also expressed in higher quantities, however differences were not statistically significant (Fig. 3). Expression of this miRNA panel was further analyzed in samples of synovial sarcoma patients in complete remission (n = 10). All seven miRNAs were significantly upregulated in the independent cohort of active synovial sarcoma patients compared to patients with synovial sarcoma in complete remission (Fig. 3)

**Fig. 4:**
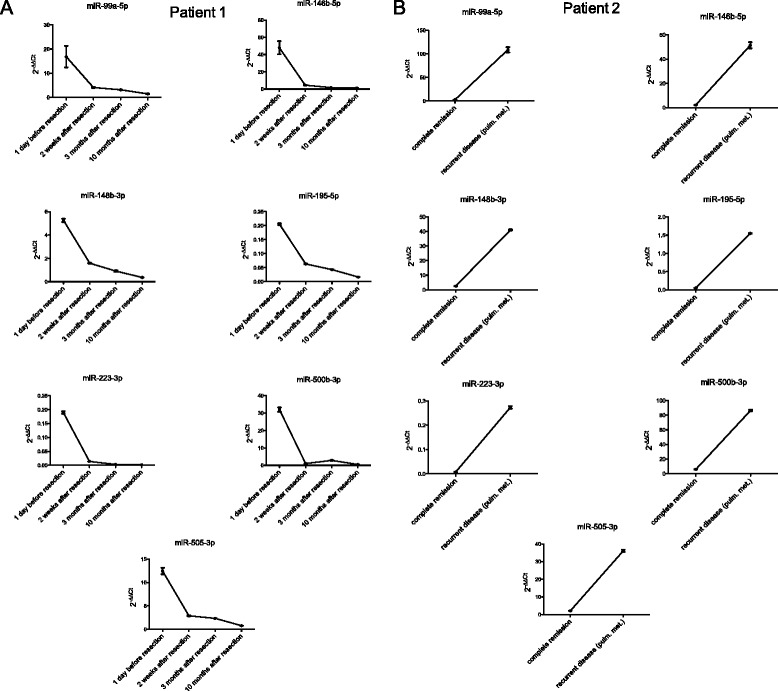
Change in miRNA-expression at remission as well as when presenting active disease in two individual synovial sarcoma patients. **a**. Patient 1 initially presented with localized disease of the lower extremity. miRNA levels were recorded 1 day before surgery and at three time-points after complete tumor resection. **b**. Patient 2 was in complete remission but developed pulmonary metastasis 4 months after the initial blood withdrawal

 To exclude a bias of fundamental expression levels of miRNAs among the individual patients with synovial sarcoma and to illustrate the change of miRNA expression at remission and in active disease, we performed a matched-pair analysis in two individual sarcoma patients. Patient 1 initially presented with localized disease of the lower extremity. The miRNA-expression before surgical resection of the tumor as well as two weeks, three months and ten months after complete surgical removal of the tumor was analyzed. The miRNA expression levels decreased rapidly after tumor resection (Fig. 4). Patient 2 was part of the synovial sarcoma in remission cohort at first with unsuspicious miRNA levels. However, she developed pulmonary metastases four months after the initial blood withdrawal. The 2nd blood withdrawal was carried out 5 month after the 1st blood withdrawal. At this time, the miRNA expression levels were significantly elevated when compared to their expression at the time of remission (Fig. 4). Thus, by performing a matched-pair analysis in these two individual sarcoma patients, we were able to illustrate the downregulation of miRNA expression at remission as well as the upregulation of the presented miRNAs in reoccurring active disease.

In summary, the detected miRNAs might provide sensitive biomarkers to distinguish patients with active synovial sarcoma from healthy controls and patients in complete remission by a simple and non-invasive blood test. They might also be useful to distinguish synovial sarcoma from other soft tissue sarcoma subtypes.

Expression profiling of miRNAs in whole blood may be altered by differences in circulating blood cells. In our two cohorts, patients with active synovial sarcoma did not differ significantly from the healthy controls, synovial sarcoma in complete remission and active leiomyosarcoma, MPNST, Ewing Sarcoma and liposarcoma group concerning age, BMI, hemoglobin-level, platelet count and leukocyte count (Additional file 1 and Additional file 2), thus showing that different miRNA expression in the patient groups evaluated was not due to changes in cell populations such as cancer- and chemotherapy-induced anemia. Furthermore, miRNA-expression levels did not differ whether patients received anticoagulation or chemotherapy. The fact that the patient receiving radiotherapy showed the lowest miRNA-expression levels of all active synovial sarcoma patients for most of the detected miRNAs (Additional file 4) was probably due to the fact that this patient was also the only patient presenting with localized disease, thus suggesting that high miRNA expression levels reflect the dissemination or possibly the activity of the disease. When analyzing miRNA expression levels in synovial sarcoma tissue and whole blood of patients with active synovial sarcoma, the majority of the detected miRNAs did not show correlations with SS18-SSX1 and 2 fusion gene phenotypes (Additional file 5). However, the sample number in these subgroups are to low to allow for a valid conclusion. A major advantage of blood sample collection in PAXgene Blood RNA Tubes is the immediate stabilization of intracellular RNA, thus preserving the gene expression profile for reliable downstream gene expression analysis. Without immediate stabilization, degradation of RNA and upregulation or downregulation of transcripts may alter the results of further analysis. Furthermore, blood samples collected in PAXgene Blood RNA Tubes can be safely stored or transported at room temperature for up to 72 h and at −20 °C or −70 °C for at least 50 months. This might reduce pre-analytical errors and facilitate future multi-centre studies, as immediate processing, which is for example needed for the detection of plasma biomarkers, is not necessary, thus avoiding potential changes in miRNA levels by different processing conditions [20].

**Fig. 5:**
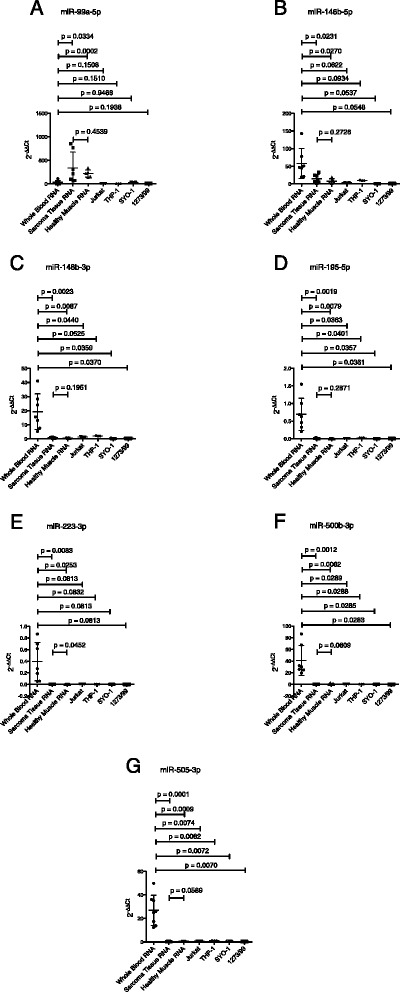
Comparison of whole blood miRNA expression levels of patients with active synovial sarcoma to corresponding synovial sarcoma tissue, healthy skeletal muscle, Jurkat (human T-lymphocyte cell line), THP-1 cells (human acute monocytic leukemia cell line) and synovial sarcoma cells (SYO-1 und 1273/99 cell lines). **a**. miR-99a-5p. **b**. miR-146b-5p. **c**. miR-148b-3p. **d**. miR-195-5p. **e**. miR-223-3p. **f**. miR-500b-3p. **g**. miR-505-3p

Keller et al., analyzing 454 whole blood samples collected in PAXgene Blood RNA Tubes from human individuals with different cancers or non-cancer diseases, were able to predict the correct disease in 67.45 % of all individuals by their miRNA expression profiles. Furthermore, they were able to distinguish lung cancer from chronic obstructive pulmonary disease (COPD) with an accuracy of 91.7 %, thus providing support for the feasibility of peripheral whole blood miRNA expression patterns in spite of the fact that changes in cell populations may affect overall miRNA profiles. They also found that only a few of the miRNAs deregulated in blood were also previously reported as deregulated in solid tissues derived from individuals with the same diseases [21], which explains why the miRNAs found to be deregulated in whole blood of synovial sarcoma patients in our study did not correlate with previous findings of deregulated miRNAs such as miR-183, miR-200b∗, miR-375, [16] let-7e, miR-99b, and miR-125-3p, [18] miR-200b, miR-200c and miR-141 [15] in synovial sarcoma tissue. This might be due to the fact that the miRNA expression patterns found in whole blood depict the miRNA expression patterns of peripheral blood mononuclear cells (PBMC) rather than the miRNA expression patterns of circulating tumor cells, as Mookherjee et al. proved in their study [22]. Furthermore, Leidinger et al. showed that especially the miRNA signature of cells of the innate immune system allowed separation between lung cancer patients and healthy controls, however pointing out that cancer-specific miRNA expression of whole blood samples are not determined by a single cell type but influenced by other blood constituents such as platelets or erythrocytes [23]. These studies point out that the miRNA signature of synovial sarcoma detected in our study represents a response of the innate immune system to the tumor rather than the miRNA expression profile of the tumor itself, thus explaining why none of the presented miRNAs match the highly expressed miRNAs in synovial sarcoma tissues as described by Fujiwara et al. [24], Hisaoka et al. [18] and Renner et al. [16]. This is further suggested by the following findings: When comparing the expression of the detected miRNAs in whole blood RNA of patients with active synovial sarcoma with the corresponding tumor tissue (n = 7), we found that most miRNAs were significantly higher expressed in whole blood RNA of patients with active synovial sarcoma (Fig. 5). MiR-99a-5p was the only miRNA being higher expressed in synovial sarcoma tissue than in whole blood. Comparing the miRNA expression levels in synovial sarcoma tissue (n = 7) with healthy skeletal muscle (n = 5), we found no significantly different expression of most of the detected miRNAs (Fig. 5). Only MiR-223-3p was significantly higher expressed in healthy skeletal muscle tissue (Fig. 5e). When comparing the miRNA expression levels of lymphocytes (Jurkat; human T lymphocyte cell line) and monocytes (THP-1; human acute monocytic leukemia cell line) to synovial sarcoma cells (SYO-1 und 1273/99 cell line), we found that most miRNAs were significantly higher expressed in human lymphocytes compared to SYO-1 and 1273/99 synovial sarcoma cells and human monocytes compared to SYO-1 and 1273/99 synovial sarcoma cells (Additional file 6). Interestingly, miR-99a-5p is the only miRNA being higher expressed both in synovial sarcoma tissue compared to whole blood cells (Fig. 5a) and synovial sarcoma cells compared to THP-1 and Jurkat cells (Additional file 6A), thus indicating that the high expression levels of miR-99a-5p in synovial sarcoma patients might not result from a miRNA expression shift of peripheral blood cells but might actually be related to the tumor itself. However, miR-99a-5p shows overlapping expression values in whole blood of patients with active synovial sarcoma compared to the different control groups (Figs. 2 and 3), thus it is not an ideal biomarker for synovial sarcoma. Furthermore, it was shown that human macrophages transfer miR-223 to hepatocarcinoma cells through intercellular contact, leading to decreased expression of stathmin-1 and insulin-like growth factor-1 receptor and decreased proliferation of tumor cells [25], which adds to the point that the detected upregulation of miR-223-3p in whole blood cells might primarily be a defense of circulating mononuclear cells against the proliferation of synovial sarcoma.

Interestingly, we found that four of the detected seven miRNAs seem to interact with the nuclear factor kappa B pathway (NF- κB), thus possibly playing an important role in toll like and cytokine receptor signaling. MiRNAs are well described regulators of the immune response [26]. The expression of miR-146b has been both shown to be upregulated by inflammation-related induction of NF-κB through a MyD88-dependent pathway, and also downregulating TNF receptor-associated factor 6 (TRAF6) and IL-1 receptor-associated kinase 1 (IRAK1), thus reducing the activity of the pathway by a negative feedback regulation loop and maintaining the balance of toll like and cytokine receptor signaling [27]. In consistency with this study, Hulsmans et al. showed that silencing of miR-146b-5p resulted in more NFκB p65 DNA binding activity and TNFα. Moreover, low levels of miR-146b-5p in monocytes of obese persons have been shown to be associated with increased mitochondrial reactive oxygen species (ROS) [28], thus showing that miR-146b inhibits NF-κB – mediated inflammation. An upregulation of miR-146b-5p might thus reflect an immune evasion of the tumor, leading to increased tumor proliferation and progression. MiR-195 also seems to interact with NF-κB, as it was shown to decrease the expression of multiple NF-κB downstream effectors [29]. Another of the NFκ-B related miRNAs is miR-223; as the transcriptional activity of miR-223 promoter was shown to be activated by Notch and NF-κB [30]. Finally, miR-500 was shown to induce gastric cancer cell proliferation and tumorigenicity by activating NF-κB. [31]. Thus, although finding the exact biological significance of the miRNAs in tumorigenesis of synovial sarcoma is a complex matter, it seems that the miRNAs detected in our study control various activities of the immune system by interacting with different pathways such as the NFκ-B signaling pathway. This further underlines our hypothesis that the detected miRNA expression levels reflect changes of the immune system as a defense to tumor progression as well as immune evasion mechanisms of the tumor.

Biomarkers for synovial sarcoma are urgently needed to identify patients with local recurrence and metastatic disease, as imaging studies are often inconclusive. Although validated miRNAs were not always significantly different in patients with active disease compared to the different control groups, miR-146b-5p, miR-500b-3p and miR-505-3p allow discrimination of patients of active synovial sarcoma patients from the different control groups, showing clear cut-off-values at approximately 18.5 (miR-146b-5p), 17.5 (miR-500b-3p) and 11.5 (miR-505-3p) 2^-ΔΔ*Ct*^; while miR-99-5p, miR-148b-3p, miR-195-5p and miR-223-3p show overlapping expression values. These miRNAs are promising candidates as diagnostic biomarkers for synovial sarcoma, potentially allowing the detection of local recurrence or distant metastasis by a simple blood test: A rise of these miRNAs beyond the cut-off value - while initially lying underneath the cut-off value following complete removal of the tumor - might indicate local recurrence or distant metastasis, as shown in Fig. 4b (Patient 2). Moreover, these miRNAs could be applied in order to distinguish between synovial sarcoma and other types of sarcoma, which may be of therapeutic importance if chemosensitive sarcoma such as the rare extraosseous Ewing’s sarcoma with spindle cell sarcoma-like pattern, often showing histological overlap with synovial sarcoma [32], are considered in differential diagnosis. Although our results proved to be highly significant, the main limitation of this study consists in the low number of patients within the different study groups. Nonetheless, one has to take into account that soft tissue sarcoma is a rare malignancy which is furthermore divided into a variety of different subtypes, thus making the collection of a large number of patients within one study group difficult. We preferred to identify a miRNA panel in a small group of patients with synovial sarcoma as an initial “proof of concept” rather than including a large number of patients with different sarcoma subtypes. As synovial sarcoma is characterized by the presence of the SS18-SSX fusion gene, which can be detected by fluorescence in situ hybridization (FISH) of tumor tissue, this sarcoma subtype can be specifically diagnosed without the risk of being confounded with other sarcoma subtypes. Through this approach, we achieved highly significant miRNA array and qRT-PCR results and a clear separation of sarcoma samples from control samples by hierarchical clustering. The fact that the miRNA signature established in this pilot study even distinguishes synovial sarcoma from leiomyosarcoma, MPNST, Ewing sarcoma and liposarcoma samples further elucidates the specificity of our results.

Further studies are warranted to validate the panel of deregulated miRNAs in a larger cohort of independent synovial sarcoma patients and to identify miRNA signatures of other sarcoma subtypes. Moreover, the existing group of patients with synovial sarcoma in complete remission needs to be closely monitored in order to evaluate a possible rise of the established miRNAs with local recurrence or distant metastasis.

## Conclusion

 Our results have identified a specific whole blood miRNA signature that may serve as an independent biomarker for the diagnosis of local recurrence or distant metastasis of synovial sarcoma and that even distinguishes synovial sarcoma from other histological sarcoma subtypes.

## Methods

### Study population

All patients included in the study were patients receiving treatment from specialists in the interdisciplinary tumor board of the Comprehensive Cancer Center Freiburg (CCCF). Patients with a history of cancer other than sarcoma or any type of systemic inflammatory disease or autoimmune disorder were excluded. The control group included healthy adults matched to the active synovial sarcoma group in terms of age, sex and body mass index (BMI). Diagnosis of synovial sarcoma, leiomyosarcoma, MPNST, Ewing sarcoma and liposarcoma was confirmed by two independent pathologists.

### Ethics, consent and permissions

Signed informed consent was obtained from all participants, allowing analysis of blood samples and all clinical data. The Ethics Committee of the Albert-Ludwigs-University of Freiburg, Germany, approved the study. The design and performance of the study is in accordance with the Declaration of Helsinki.

### Blood sampling

All blood samples were collected by puncture of the antecubital vein without tourniquet through a 20-gauge needle. The first 3 ml of blood were discarded. Each 2.5 ml of whole blood were collected and stabilized in PAXgene Blood RNA Tubes (PreAnalytiX, Hombrechtikon, Switzerland). The RNA Tubes were incubated for at least 2 h at room temperature after blood collection to ensure complete lysis of blood cells and were then stored at −20 °C until further processing. Before starting the procedure, they were equilibrated to room temperature. Total RNA > 18 nucleotides (including miRNA) was purified manually using the PAXgene Blood miRNA Kit (PreAnalytiX) according to the manufacturer’s protocol.

### miRNA Array

RNA quality and quantity was assessed by capillary electrophoresis using the Fragment Analyzer and Standard/High sensitivity RNA Analysis kits (Advanced Analytical Technologies, Ames, IA, U.S.). Total whole blood RNA of patients with active synovial sarcoma (n = 5) and healthy controls (n = 5) was then analyzed using an Affymetrix GeneChip miRNA Array v. 4.0 (Affymetrix, Santa Clara, CA, U.S.). 275 ng of total RNA was labeled with Biotin using a 3DNA Array Detection FlashTag™ Biotin HSR kit (Genisphere, Hatfield, PA, U.S.) following the manufacturer’s protocol, being subsequently hybridized overnight. The GeneChip® miRNA 4.0 arrays, containing 30,424 total mature miRNA probe sets including 2.578 mature human miRNAs and miRNAs of 202 other organisms, were washed and stained using the Affymetrix GeneChip Hybridization Wash and Stain Kit and were then scanned with the Affymetrix GeneChip Scanner 3000 7G (Affymetrix, Santa Clara, CA, U.S.).

### Cell culture

The samples of the human synovial sarcoma cell lines SYO-1 und 1273/99 were a donation from Dr. Marcus Renner, Institute of Pathology, University of Heidelberg.

SYO-1 synovial sarcoma cells were cultured in Dulbecco’s modified minimal essential medium (life technologies, Carlsbad, CA, U.S.A.) supplemented with 10 % FBS superior (Biochrom, Berlin, Germany), 100 U/ml Penicillin-Streptomycin (life technologies) and 0.5 % Sodium Pyruvate (Biochrom, Berlin, Germany).

1273/99 synovial sarcoma cells were cultured in F-12 Nutrient mixture (Ham) (life technologies) supplemented with 10 % FBS superior and 100 U/ml Penicillin-Streptomycin.

THP-1 cells (human acute monocytic leukemia cell line) and Jurkat cells (human T lymphocyte cell line) were cultured in RPMI Medium 1640 (with L-Glutamine; life technologies) supplemented with 10 % FBS superior and 100 U/ml Penicillin-Streptomycin.

### RNA Isolation

Total RNA of SYO-1, 1273/99, THP-1 and Jurkat cells was purified using the miRNeasy Mini Kit (Qiagen, Hilden, Germany) according to the manufacturer’s protocol. Total RNA of tissue sample sections, each with a thickness of 10 μm and a surface area of approximately 250 mm^2^, was purified after deparaffinization using the miRNeasy FFPE Kit (Qiagen) with an extension of the time of incubation with proteinase K at 56 °C from 15 min to 60 min for higher RNA yields. The remaining steps were carried out according to the manufacturer’s protocol. The RNA was quantified using a Nanodrop 2000 (Thermo Fisher Scientific, Waltham, MA, USA).

### Preparation of cDNA

For detection of miRNA expression levels, cDNA synthesis of whole blood RNA, cell RNA and tissue RNA (275 ng) was carried out using the miScript II RT Kit (Qiagen) according to the manufacturer’s protocol. The reverse transcription reaction was incubated for 60 min at 37 °C, followed by 5 min at 95 °C to inactivate the miScript Reverse Transcriptase Mix.

For detection of the SS18-SSX1 and SS18-SSX2 fusion gene transcript in tissue samples, each 275 ng of RNA was converted to cDNA using the AffinityScript Multi Temperature cDNA Synthesis Kit (Agilent Technologies, Santa Clara, CA, USA) according to the manufacturer’s protocol. The reverse transcription reaction was incubated for 10 min at 25 °C, followed by 1 h at 42 °C and 15 min at 70 °C.

### qRT-PCR

For detection of miRNA expression levels, qRT-PCR was performed using the miScript SYBR Green PCR Kit (Qiagen, Hilden, Germany) according to the manufacturer’s protocol. Briefly, the cycling conditions consisted of an initial activation step of the HotStarTaq DNA Polymerase for 15 min at 95 °C, followed by 45 cycles of denaturation for 15 s at 94 °C, annealing for 30 s at 55 °C and extension for 30 s at 70 °C with fluorescence data collection being performed during the extension step. MiScript Primer Assays were purchased from Qiagen. *Ct*-values were normalized to RNU6-2B, a frequently used reference gene [33] and 2^-ΔΔ*Ct*^ values [34] were analyzed using Student’s *t*-test for independent samples.

For detection of the SS18-SSX1 and SS18-SSX2 fusion gene transcript in tissue samples, qRT-PCR was performed using the Absolute qPCR ROX Mix (ThermoScientific, Waltham, MA, USA) and the following primers: SS18-SSX1 + FAM (Hs 03024820_ft), SS18-SSX2 + FAM (Hs03024398_ft) (TaqMan Gene Expression Assays; Applied Biosystems by life technologies, Carlsbad, CA, USA) and the GAPDH-Primer Set (GAPDH-probe 899, GAPDH-875 F und GAPDH 946-R (Eurofins MWG Operon, Huntsville, AL, USA) as the internal control. Briefly, the cycling conditions were enzyme activation at 95 °C for 15 min, followed by 50 cycles of denaturation at 95 °C for 15 s and annealing/extension at 60 °C for 1 min. Ct-values < 36 were considered as positive.

### Statistics

For analysis of the miRNA array, CEL-files of the raw data were produced with Affymetrix GeneChip Command Console Software Version 4.0. (Affymetrix, Santa Clara, CA, U.S.) Partek Genomics Suite software (Version 6.14.0923; Partek, Inc., St. Louis, MO, USA) was used for further analysis. CEL-files were imported including control and interrogating probes, and the arrays were normalized using quantile normalization. Probeset summarization was done using Median Polish. Probe values were log2 transformed. In order to detect differential miRNA expression between the 2 groups, 1-way ANOVA was performed [35] and Fisher’s Least Significant Difference (LSD) was used as contrast method.

MultiExperiment Viewer 4.8 was used for hierarchical clustering of miRNA array data [36]. 2^-ΔΔ*Ct*^ and patients’ demographic data and blood counts were compared using Student’s *t*-test for independent samples. p-values were rounded to 4 significant digits; p-values below 0.05 were considered statistically significant.

## Additional files

Additional file 1:
**Demographic patient data and blood count of patients with active sarcoma and control groups.** Demographic patient data (age, BMI) and blood count (hemoglobin level, platelet count and leukocyte count) of patients with active synovial sarcoma compared to healthy donors, patients with synovial sarcoma in remission and patients with active leiomyosarcoma, MPNST, Ewing sarcoma and liposarcoma. Data are presented as mean value ± standard error of mean (SEM). p- values were determined using a Student’s *t*-test for independent samples. Hb = Hemoglobin. BMI = Body Mass Index. (PPTX 68 kb) Additional file 2:
**Demographic patient data and blood count of patients with active sarcoma (Individual Cohort (IC)) and control groups.** Demographic patient data (age, BMI) and blood count (hemoglobin level, platelet count and leukocyte count) of patients with active synovial sarcoma (Individual Cohort (IC)) compared to healthy donors and patients with synovial sarcoma in remission. Data are presented as mean value ± standard error of mean (SEM). p- values were determined using a Student’s *t*-test for independent samples. Hb = Hemoglobin. BMI = Body Mass Index. IC = Independent Cohort. (PPTX 66 kb) Additional file 3:
**Disease and therapy status of sarcoma patients. **M_0_ = localized disease. M_1_ = metastatic disease. Current chemotherapy/radiotherapy involves treatment within the last 6 weeks. (PPTX 67 kb) Additional file 4:
** Illustration of the miRNA-expression levels of the two active synovial sarcoma patients who received chemotherapy (Patient 2 and 3 of the screening cohort of active synovial sarcoma patients as depicted in Additional file** **3** **) marked with a triangle, patients receiving anticoagulation (Patient 3 of the screening cohort of active synovial sarcoma patients and Patient 1 of the independent cohort of active synovial sarcoma patients as depicted in Additional file** **3** **) marked in green and patients receiving radiotherapy/presenting localized disease (Patient 1 of the screening cohort of active synovial sarcoma patients as depicted in Additional file** **3** **) marked in red.** A, C, E, G, I, K and M include the screening cohort of active synovial sarcoma patients, B, D, F, H, J, L and N depict the independent cohort (IC) of synovial sarcoma patients. (JPEG 5044 kb) Additional file 5:
**Comparison of whole blood miRNA expression levels of patients with active synovial sarcoma and miRNA expression levels of corresponding synovial sarcoma tissue.** MiRNA-expression of patients with synovial sarcoma expressing SS18-SSX2 are marked in purple. A. miR-99a-5p. B. miR-146b-5p. C. miR-148b-3p. D. miR-195-5p. E. miR-223-3p. F. miR-500b-3p. G. miR-505-3p. (JPEG 2837 kb) Additional file 6:
**MiRNA expression levels of Jurkat (human T-lymphocyte cell line) and THP-1 cells (human acute monocytic leukemia cell line) compared to synovial sarcoma cells (SYO-1 und 1273/99 cell lines). A.** miR-99a-5p. B. miR-146b-5p. C. miR-148b-3p. D. miR-195-5p. E. miR-223-3p. F. miR-500b-3p. G. miR-505-3p. (JPEG 2926 kb)

## Abbreviations

BMI: Body mass Index
CT: Computed Tomography
FISH: Fluorescence In Situ Hybridization
Hb: Hemoglobin
MiRNA: MicroRNA
MPNST: Malignant Peripheral Nerve Sheath Tumor
MRI: Magnetic Resonance Imaging
NF-κB: Nuclear factor κ B
qRT-PCR: quantitative Reverse Transcription - Polymerase Chain Reaction
