# Supplementary material for: Identification of a blood-borne miRNA signature of synovial sarcoma
Source: Mol Cancer. 2015 Aug 7;14:151. doi: 10.1186/s12943-015-0424-z (PMC4528907; doi:10.1186/s12943-015-0424-z)
Supplement: Additional file 1: — Demographic patient data and blood count of patients with active sarcoma and control groups. Demographic patient data (age, BMI) and blood count (hemoglobin level, platelet count and leukocyte count) of patients with active synovial sarcoma compared to healthy donors, patients with synovial sarcoma in remission and patients with active leiomyosarcoma, MPNST, Ewing sarcoma and liposarcoma. Data are presented as mean value ± standard error of mean (SEM). p- values were determined using a Student’s t-test for independent samples. Hb = Hemoglobin. BMI = Body Mass Index. (PPTX 68 kb) [file 12943_2015_424_MOESM1_ESM.pptx]

## Slide 1
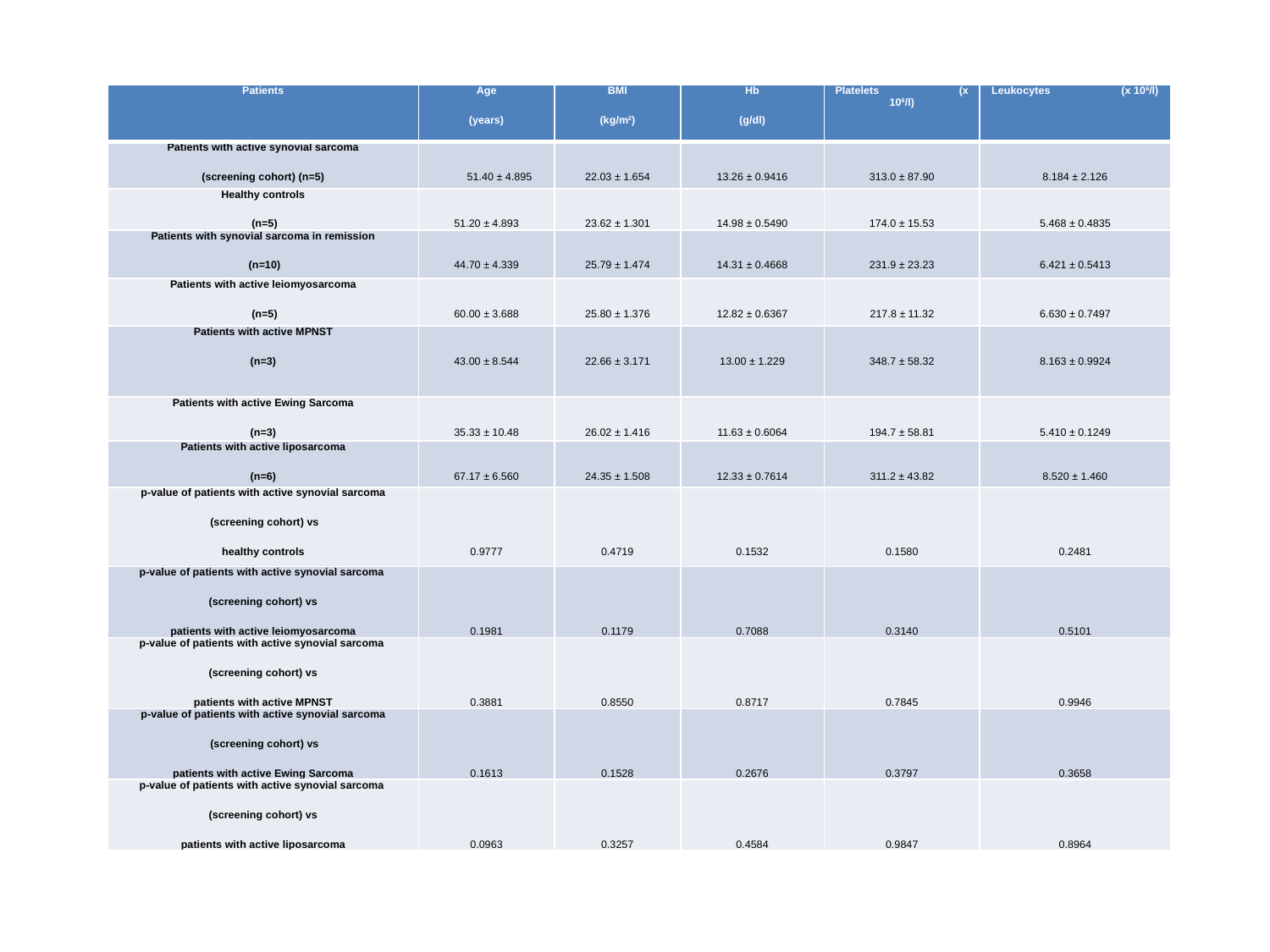

| Patients | Age (years) | BMI (kg/m2) | Hb (g/dl) | Platelets (x 106/l) | Leukocytes (x 106/l) |
| --- | --- | --- | --- | --- | --- |
| Patients with active synovial sarcoma (screening cohort) (n=5) | 51.40 ± 4.895 | 22.03 ± 1.654 | 13.26 ± 0.9416 | 313.0 ± 87.90 | 8.184 ± 2.126 |
| Healthy controls (n=5) | 51.20 ± 4.893 | 23.62 ± 1.301 | 14.98 ± 0.5490 | 174.0 ± 15.53 | 5.468 ± 0.4835 |
| Patients with synovial sarcoma in remission (n=10) | 44.70 ± 4.339 | 25.79 ± 1.474 | 14.31 ± 0.4668 | 231.9 ± 23.23 | 6.421 ± 0.5413 |
| Patients with active leiomyosarcoma (n=5) | 60.00 ± 3.688 | 25.80 ± 1.376 | 12.82 ± 0.6367 | 217.8 ± 11.32 | 6.630 ± 0.7497 |
| Patients with active MPNST (n=3) | 43.00 ± 8.544 | 22.66 ± 3.171 | 13.00 ± 1.229 | 348.7 ± 58.32 | 8.163 ± 0.9924 |
| Patients with active Ewing Sarcoma (n=3) | 35.33 ± 10.48 | 26.02 ± 1.416 | 11.63 ± 0.6064 | 194.7 ± 58.81 | 5.410 ± 0.1249 |
| Patients with active liposarcoma (n=6) | 67.17 ± 6.560 | 24.35 ± 1.508 | 12.33 ± 0.7614 | 311.2 ± 43.82 | 8.520 ± 1.460 |
| p-value of patients with active synovial sarcoma (screening cohort) vs healthy controls | 0.9777 | 0.4719 | 0.1532 | 0.1580 | 0.2481 |
| p-value of patients with active synovial sarcoma (screening cohort) vs patients with active leiomyosarcoma | 0.1981 | 0.1179 | 0.7088 | 0.3140 | 0.5101 |
| p-value of patients with active synovial sarcoma (screening cohort) vs patients with active MPNST | 0.3881 | 0.8550 | 0.8717 | 0.7845 | 0.9946 |
| p-value of patients with active synovial sarcoma (screening cohort) vs patients with active Ewing Sarcoma | 0.1613 | 0.1528 | 0.2676 | 0.3797 | 0.3658 |
| p-value of patients with active synovial sarcoma (screening cohort) vs patients with active liposarcoma | 0.0963 | 0.3257 | 0.4584 | 0.9847 | 0.8964 |
