# Supplementary material for: Identification of a blood-borne miRNA signature of synovial sarcoma
Source: Mol Cancer. 2015 Aug 7;14:151. doi: 10.1186/s12943-015-0424-z (PMC4528907; doi:10.1186/s12943-015-0424-z)
Supplement: Additional file 2: — Demographic patient data and blood count of patients with active sarcoma (Individual Cohort (IC)) and control groups. Demographic patient data (age, BMI) and blood count (hemoglobin level, platelet count and leukocyte count) of patients with active synovial sarcoma (Individual Cohort (IC)) compared to healthy donors and patients with synovial sarcoma in remission. Data are presented as mean value ± standard error of mean (SEM). p- values were determined using a Student’s t-test for independent samples. Hb = Hemoglobin. BMI = Body Mass Index. IC = Independent Cohort. (PPTX 66 kb) [file 12943_2015_424_MOESM2_ESM.pptx]

## Slide 1
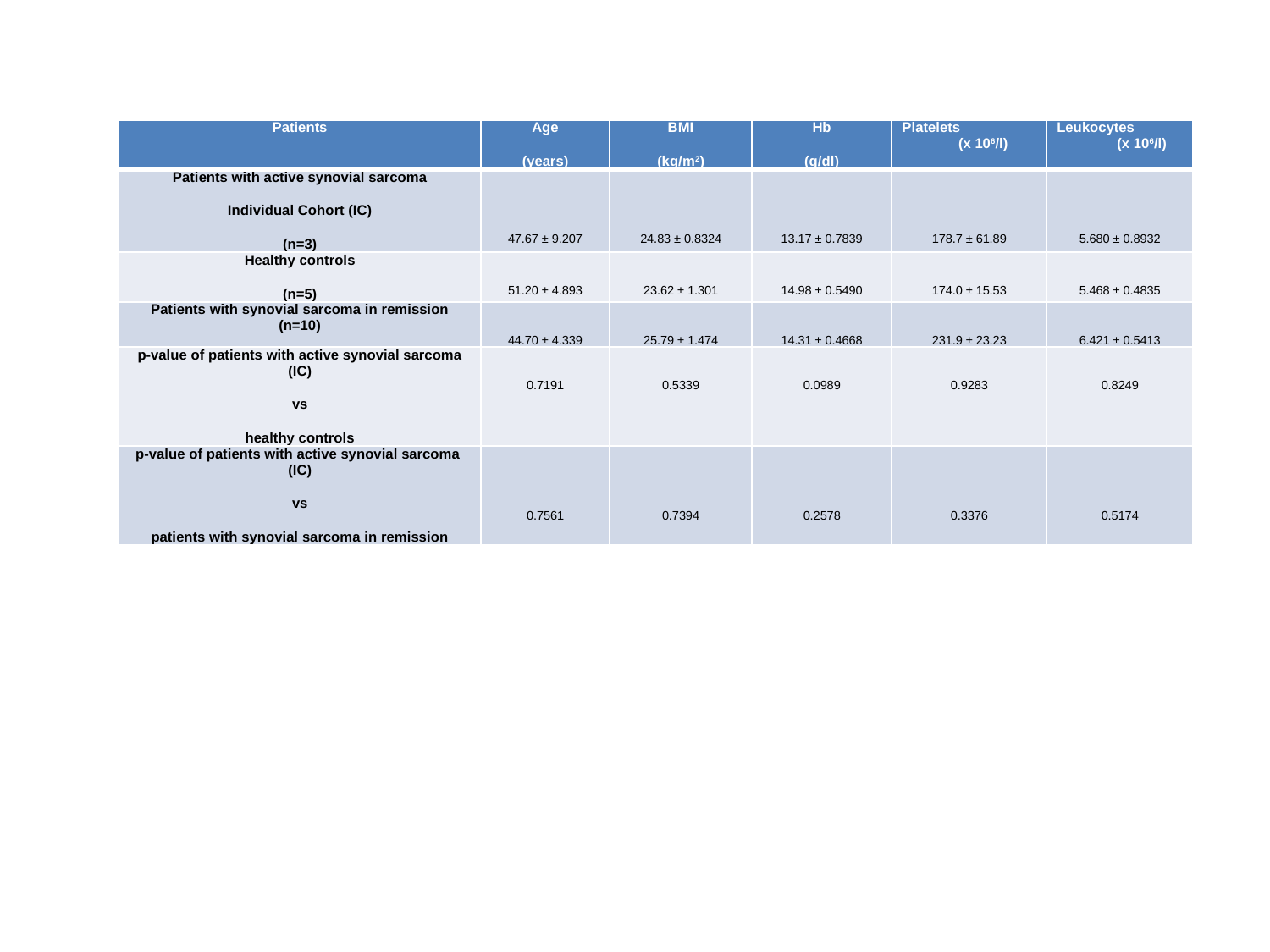

| Patients | Age (years) | BMI (kg/m2) | Hb (g/dl) | Platelets (x 106/l) | Leukocytes (x 106/l) |
| --- | --- | --- | --- | --- | --- |
| Patients with active synovial sarcoma Individual Cohort (IC) (n=3) | 47.67 ± 9.207 | 24.83 ± 0.8324 | 13.17 ± 0.7839 | 178.7 ± 61.89 | 5.680 ± 0.8932 |
| Healthy controls (n=5) | 51.20 ± 4.893 | 23.62 ± 1.301 | 14.98 ± 0.5490 | 174.0 ± 15.53 | 5.468 ± 0.4835 |
| Patients with synovial sarcoma in remission (n=10) | 44.70 ± 4.339 | 25.79 ± 1.474 | 14.31 ± 0.4668 | 231.9 ± 23.23 | 6.421 ± 0.5413 |
| p-value of patients with active synovial sarcoma (IC) vs healthy controls | 0.7191 | 0.5339 | 0.0989 | 0.9283 | 0.8249 |
| p-value of patients with active synovial sarcoma (IC) vs patients with synovial sarcoma in remission | 0.7561 | 0.7394 | 0.2578 | 0.3376 | 0.5174 |
