# Supplementary material for: Identification of a blood-borne miRNA signature of synovial sarcoma
Source: Mol Cancer. 2015 Aug 7;14:151. doi: 10.1186/s12943-015-0424-z (PMC4528907; doi:10.1186/s12943-015-0424-z)
Supplement: Additional file 3: — Disease and therapy status of sarcoma patients. M0 = localized disease. M1 = metastatic disease. Current chemotherapy/radiotherapy involves treatment within the last 6 weeks. (PPTX 67 kb) [file 12943_2015_424_MOESM3_ESM.pptx]

## Slide 1
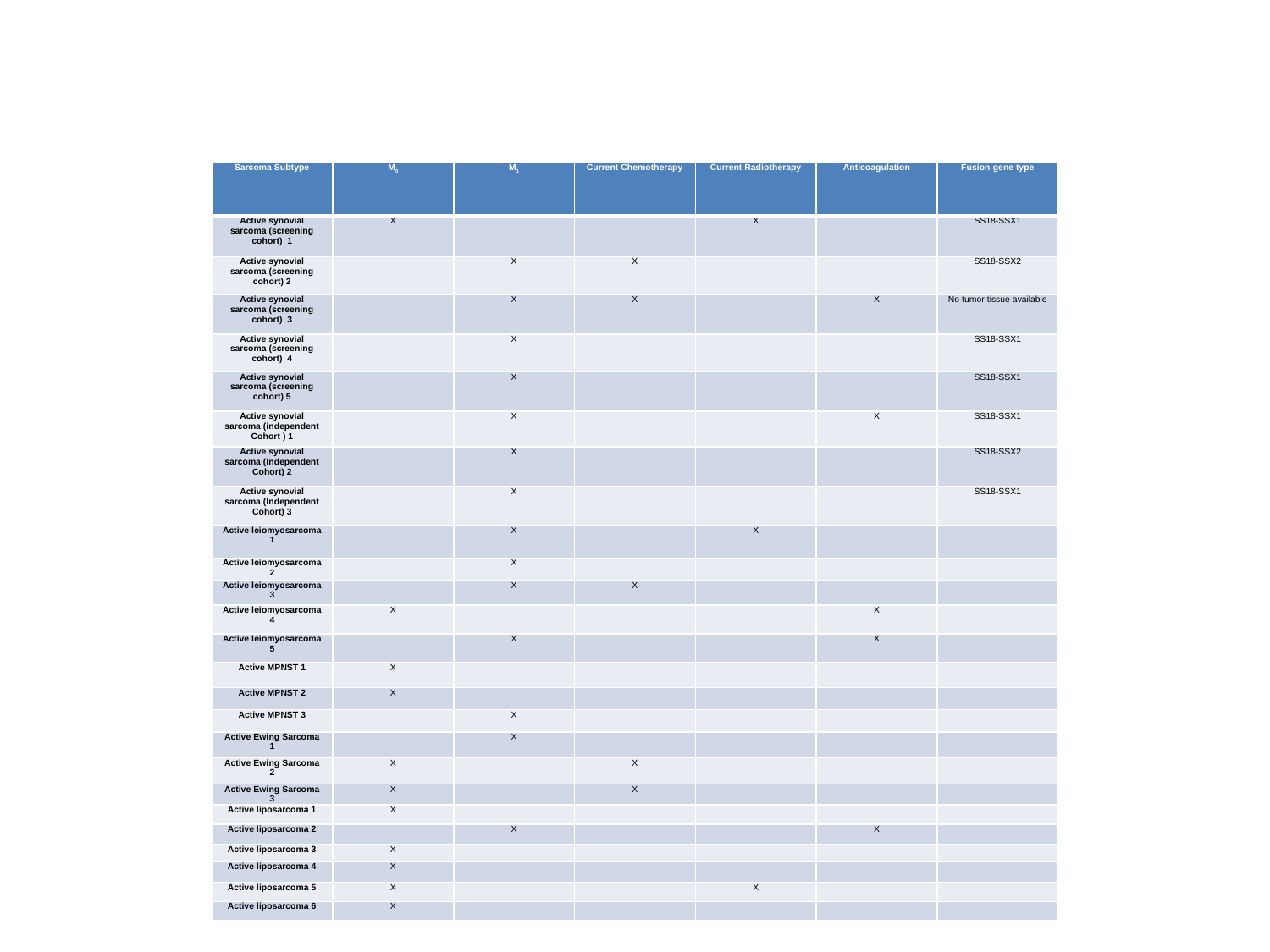

| Sarcoma Subtype | M0 | M1 | Current Chemotherapy | Current Radiotherapy | Anticoagulation | Fusion gene type |
| --- | --- | --- | --- | --- | --- | --- |
| Active synovial sarcoma (screening cohort) 1 | X | | | X | | SS18-SSX1 |
| Active synovial sarcoma (screening cohort) 2 | | X | X | | | SS18-SSX2 |
| Active synovial sarcoma (screening cohort) 3 | | X | X | | X | No tumor tissue available |
| Active synovial sarcoma (screening cohort) 4 | | X | | | | SS18-SSX1 |
| Active synovial sarcoma (screening cohort) 5 | | X | | | | SS18-SSX1 |
| Active synovial sarcoma (independent Cohort ) 1 | | X | | | X | SS18-SSX1 |
| Active synovial sarcoma (Independent Cohort) 2 | | X | | | | SS18-SSX2 |
| Active synovial sarcoma (Independent Cohort) 3 | | X | | | | SS18-SSX1 |
| Active leiomyosarcoma 1 | | X | | X | | |
| Active leiomyosarcoma 2 | | X | | | | |
| Active leiomyosarcoma 3 | | X | X | | | |
| Active leiomyosarcoma 4 | X | | | | X | |
| Active leiomyosarcoma 5 | | X | | | X | |
| Active MPNST 1 | X | | | | | |
| Active MPNST 2 | X | | | | | |
| Active MPNST 3 | | X | | | | |
| Active Ewing Sarcoma 1 | | X | | | | |
| Active Ewing Sarcoma 2 | X | | X | | | |
| Active Ewing Sarcoma 3 | X | | X | | | |
| Active liposarcoma 1 | X | | | | | |
| Active liposarcoma 2 | | X | | | X | |
| Active liposarcoma 3 | X | | | | | |
| Active liposarcoma 4 | X | | | | | |
| Active liposarcoma 5 | X | | | X | | |
| Active liposarcoma 6 | X | | | | | |
